# Experimental study on the single basin solar still integrated with shell and spiral finned tube latent heat storage system enhanced by copper oxide nanoparticles

**DOI:** 10.1007/s11356-022-24104-3

**Published:** 2022-11-16

**Authors:** Saleh Shalaby, Abd Elnaby Kabeel, Bahaa Eddin Moharram, Areeg Shama, Hatem Abosheiasha Abosheiasha

**Affiliations:** 1 Engineering Physics and Mathematics Department, Faculty of Engineering, Tanta University, Tanta, 31733 Egypt; 2 Mechanical Power Engineering Department, Faculty of Engineering, Tanta University, Tanta, 31733 Egypt; 3 Faculty of Engineering, Delta University for Science and Technology, Gamasa, Egypt

**Keywords:** Solar still, Water distillation, PCM, Paraffin wax, Nanomaterial, Thermal storage

## Abstract

In this work, a latent heat storage system was designed, installed, and tested when it was integrated with a single basin solar still. The latent heat storage system is a shell and spiral finned tubes, where 20 kg of a paraffin-CuO nanocomposite with a weight fraction concentration of 1% was poured into the shell while hot water from the solar collector was being pumped through the spiral finned tubes. In order to identify the effect of implementing the storage system on the performance of the solar still, two experiments were conducted, with and without storage, under approximatelysimilar weather conditions. The proposed design of the storage system succeeded to overcome all challenges associated with using paraffin wax as storage material with the solar still. The obtained results revealed that an improvement in fresh water daily productivity of 4.54% was achieved when the storage system was integrated with the solar still. The economic analysis showed that the cost per 1 L of fresh water was 0.25 $/L when the storage unit was used. This high cost will be significantly reduced when a large-scale system is installed.

## Introduction

The single basin solar still (SBSS) is considered the simplest system used for low-capacity water destination (Shalaby 2017). Many designs were introduced to improve the productivity of the SBSS, such as using finned (Velmurugan et al. 2008a, b; Panchal et al. 2022) and v-corrugated (Omara et al. 2011; El-Sebaii and Shalaby 2015) absorber, double slope glass cover (Kalidasa Murugavel et al. 2010; Shoeibi et al. 2022), and reflecting mirror (Shanmugan et al. 2008; Tiwari and Suneja 1998). Before at least 10 years, many sensible heat storage materials were used with the SBSS, such as black matt rubber (Akash et al. 1998), black gravel (Nafey et al. 2001; Sakthivel and Shanmugasundaram 2008), charcoal (Naim and Abd El Kawi 2003; Okeke et al. 1990), and black rocks (Abdallah et al. 2009). Nowadays, sensible heat storage is rarely used with solar stills due to its low energy storage density. On the other hand, the latent heat storage system has two advantages: first, it has high energy storage density and can discharge the stored energy at an approximately constant temperature. The different types of phase change materials (PCM) used in these storage systems were reviewed by Zayed et al. (2019). They found that utilization of paraffin wax (PW) as a PCM in solar thermal applications is one of the most recognized research noticed in the last decade. Implementing PW as a storage system within the SBSS is also of great interest in recent water desalination research.

A mathematical model of the SBSS with PW as PCM was prepared by Ansari et al. (2013). They used three types of PW of melting points equaling to 56, 52, and 42 °C as PCM under the SBSS absorber plate. They found that the SBSS productivity increases by 40% when the PW with a melting point 56 or 52 °C is used as PCM while it increases by 8% when the PW of 42 °C is used compared to the system free of PCM.

Kabeel and Abdelgaied (Zayed et al. 2019) experimentally studied the SBSS with and without PCM. Paraffin wax with melting point 56 °C is used as PCM under the absorber plate. They found that the fresh water productivity of the SBSS with and without the PCM is 7.54 and 4.51 L/m^2^.day, respectively**.** Yousef and Hassan (Yousef and Hassan 2019a, b) experimentally studied the SBSS integrated with 17 kg of PW with a melting point ranging from 56 to 58 °C. In addition to the conventional still, downward pin fins or black steel mesh fibers were used to improve the heat transfer from/to the PCM. They found that the SBSS with pin fins is the best among their studied systems as its productivity was enhanced by 16.8% compared to the conventional solar still. Recently, the v-corrugated and downward finned plate solar still integrated with PW as PCM was experimentally studied by Hammad et al. (Hammad et al. 2022). Their results showed that the distiller with v-corrugated plate and PCM maximized energo-economic performance and freshwater production.

An SBSS integrated with PW with a melting point of 56 °C and heated by a PV/T collector was theoretically studied by Hedayati-Mehdiabadi et al. (Hedayati-Mehdiabadi et al. 2020). Their proposed system produces 13 kg/day during summer when 30 kg of PW was used under the absorber plate of 2 m^2^. To improve the performance of the SBSS integrated with PW, a PV module was used by Abd Elbar and Hassan (Elbar and Hassan 2019). The PV provides the power needed to heat saline water and is also used as a reflector. They found that their proposed system increases the productivity by 11.7% compared to the conventional solar still. At the same time, this improvement reached 19.4 when an air fan was used to cool the PV and the solar still glass cover.

In order to solve the problem of volume boost upon PW melting, a new design of the SBSS with a v-corrugated absorber was introduced by Shalaby et al. (Shalaby et al. 2016). Their design allows the molten paraffin to expand and flow through tubes from the PCM storage tank to an external reservoir. Their proposed system was tested when 18 kg of PW with a melting pint of 56 °C was used as the PCM. For comparison purpose, their system was also tested without the PCM. They concluded that the productivity of their system is improved by 12% when the PCM was used.

The stepped solar still was experimentally studied with and without a PCM by Tabrizi (Tabrizi et al. 2010). The stills consist of 15 stepped absorber plates covered with inclined glass on the top with an air gap of about 2.5 cm. A weir on each step was used to force the saline water to zigzag through the absorber plate. They used PW with a melting temperature of 57 °C as the PCM beneath the stepped absorber. They found that the productivity of the still without the PCM is slightly higher than the still with the PCM during sunny days, while during the partially cloudy days the still with the PCM is superior to that without the PCM as its productivity is 3.4 L/m^2^day compared to the 2.1 L/m^2^day for the still without the PCM. So, they recommended the still without the PCM for sunny regions while the still with the PCM was recommended for partially cloudy regions. A similar stepped solar still was theoretically studied by Sarhaddi et al. (Sarhaddi et al. 2017). Eighteen kg of PW with a melting point of 56 °C was used as the PCM under a stepped absorber. Their design also includes weirs at the end of each step, as discussed by Tabrizi (Tabrizi et al. 2010). To validate their theoretical model, the obtained results were compared to that experimentally obtained by Tabrizi et al. (Tabrizi et al. 2010). During sunny days, they found that the daily productivity of the still without PCM is 7.05 L/m^2^ compared to the 6.63 L/m^2^ for the still with PCM, whereas during semi-cloudy days the daily productivities of the still with and without PCM are 4.94 and 3.84 L/m^2^, respectively. The above results show that a significant improvement in daily productivity was achieved when the PCM was used during the semi-cloudy days (Tabrizi et al. 2010; Sarhaddi et al. 2017).

A concentrator is used by Arunkumar et al. (Arunkumar et al. 2013) to increase the solar radiation incident on a hemispherical basin solar still (HBSS) integrated with a PCM. The solar concentered and is fixed under the HBSS. Copper balls with a 2.8-cm diameter filled and with commercial-grade PW as the PCM (melting point = 58–60 °C) are placed in the still water. Their experimental results showed that the productivity increases from 3.52 to 4.46 L/m^2^/day when the PCM is used. Kabeel and Abdelgaied (Kabeel and Abdelgaied 2017) experimentally studied the SBSS integrated with PW as the PCM. In their system, the basin water is heated by the solar radiation incident on the still and by a parabolic concentrator as well. The oil heat exchanger transferred the heat from the parabolic concentrator to the basin water. They used PW with a melting temperature of 56 °C as the PCM poured in fiberglass tank fixed at the still bottom. The daily productivity of their proposed system reached 10.77 L/m^2^/day.

The reviewed results presented in this work reveal that PW is commonly used as a thermal storage material within solar stills. The reviewed results also showed that a significant improvement is achieved when the thermal storage system is implemented with the solar still. The results also showed that the PCM is commonly used under the absorber of the solar still basin in most of the literature studies. So, many challenges were found in this location, such as the limited amount of the PCM that can be used and the PCM leakage upon the melting process. In addition, there is limited options in designs aimed to improve the heat transfer from/to the PCM. So, in this work, a separate latent heat storage system (LHSS) was used with the SBSS for the first time, as far as the authors know, to overcome all difficulties associated with a solar still with built-in PCM. The separate LHSS introduces a perfect solution for the PCM leakage problem and provides high energy storage capacity. In addition,it provides unlimited design options to improve the heat transfer, such as using spiral fins that were implemented in this work. Moreover, nanoparticles of CuO are safely used to improve the thermal conductivity of paraffin wax without any scare from its leakage. The LHSS was integrated with the SBSS and tested under the prevailing weather of Tanta city.

## Experimental work

The LHSS and SBSS designs and constructions are discussed in detail in this section. The experimental setup and testing procedure are also presented.

### The materials and construction

The proposed system mainly consists of three units: first, the single basin solar still, the LHSS, and a solar water heater (SWH). The LHSS was connected to the SWH in a closed loop when high solar radiation is available, or to the SBSS at night or during periods of low solar radiation (sun fade or cloudy periods).

**Fig. 1:**
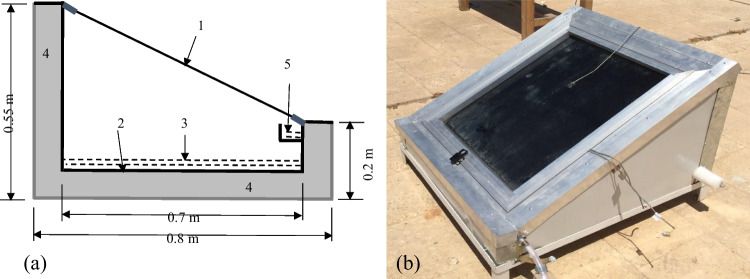
Schematic diagram of the SBSS (**a**) and photograph of the SBSS (**b**). 1, Glass cover; 2, absorber plate; 3, brackish water; 4, insulation; 5, distilled water

To avoid any corrosions, the solar still basin is made with stainless steel with a 0.7 m × 0.7 m bottom length × width and 0.55 m north and 0.2 m south heights, as shown in Fig. 1. The basin bottom and sides were insulated with 0.05 m of foam, while its upper side was covered by a transparent glass of 0.004 m thickness. The distilled water collection channel was located at the south side interior, as shown in Fig. 1a. The basin bottom was painted black while all the sides were kept without painting to act as reflectors as many researchers recommended (Tanaka 2009; Omara et al. 2013). All the still sides and bottom were shielded by painted galvanized iron sheets sandwiching a layer of foam as an insulation material.

**Fig. 2:**
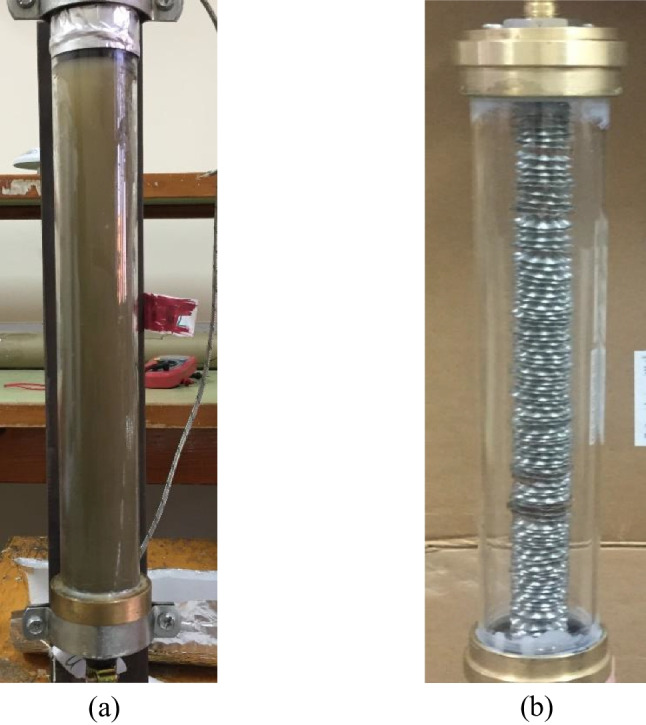
The LHSS prototype (**a**) and the spiral finned tube used in the LHSS (**b**)

In order to improve the LHSS, the thermal storage prototype (TSP) shown in Fig. 2 was first designed, constructed, and tested when the PW/CuO composite with different concentrations (0, 0.5, 1, 1.5, and 2%) was used as the PCM. The effect of using the spiral finned tube shown in Fig. 2b is also tested. More details about the prototype design and testing procedure can be found elsewhere (Shama et al. 2021).

**Fig. 3:**
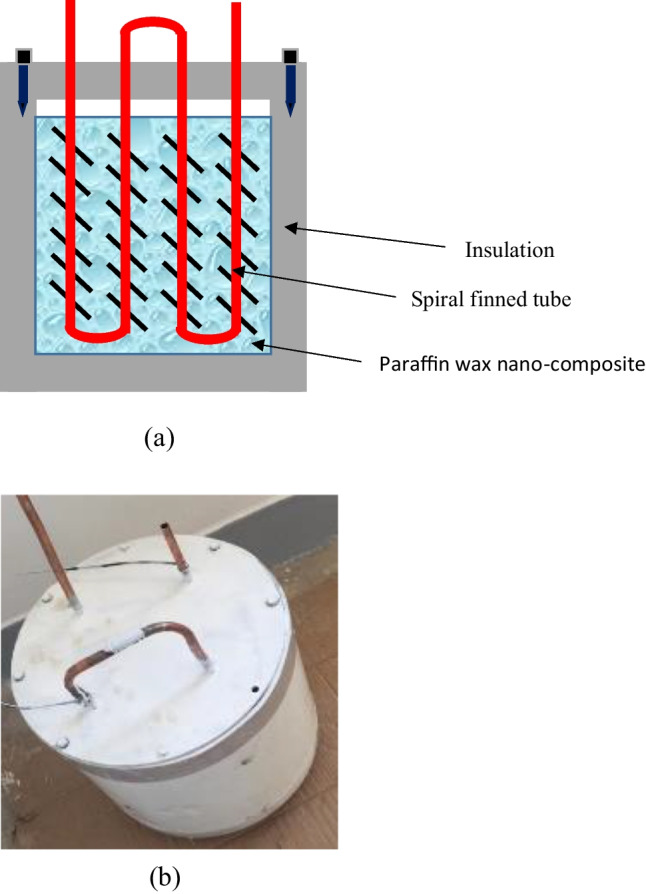
**a** Schematic diagram of the shell and spiral and finned tube storage unit and **b** photograph of the shell and spiral and finned tube storage unit

Based on the results obtained from testing the TSP, an enhanced LHSS was designed and constructed, where the latent heat storage system is a shell and spiral finned tubes. Twenty kilograms of a paraffin-CuO nanocomposite with a concentration of 1% (optimal concentration) was poured in the shell where it was heated during the charging process by the hot water that flowed through the finned tubes. The used tubes are made of copper, while the attached spiral fins were made of iron. The detailed design of the LHSS is shown in Fig. 3. The LHSS design premises easy removal of the spiral finned tube, which was designed to be a completely separate part that can be integrated with the shell from its upper using nails to ease handling, as seen in Fig. 3b. The shell is a cylinder with an internal diameter of 30 cm and made of stainless steel. It was insulated by 4 cm of foam and shielded by painted galvanized iron.

**Fig. 4:**
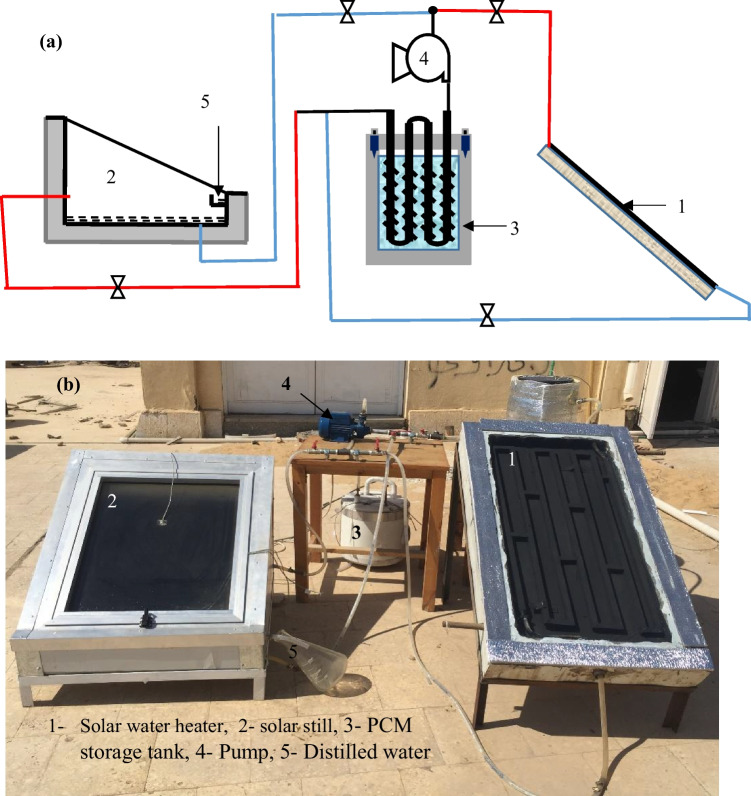
Schematic diagram (**a**) and photograph (**b**) of the solar still with enhanced PCM storage system heated by SWH

The solar water heater with an area 0.5 m^2^ was designed and constructed to provide the heat needed to completely melt the PCM nanocomposite where a flat sheet made of galvanized iron attached with rectangle copper tubes was used as the absorber, and all the sides and bottom are insulted by 5 cm of foam sandwiched by two sheets of galvanized iron. The upper side of the SWH was covered by a sheet of glass of 4 mm thickness. The water flowed inside the rectangular tubes in a closed cycle between the SWH with a water storage tank and the LHSS when abundant solar radiation was available while it was disconnected during the night or when the sun seemed to be fading. In this case, the LHSS is reconnected to the solar still using manual valves. The schematic diagram and photograph of the proposed system are exhibited in Fig. 4a and b, respectively.

### Experimental procedure

In order to identify the effect of using the LHSS on the performance of the SBSS, two experiments were conducted under approximately the same weather conditions. The first experiment was conducted without using the storage system where the conventional solar still was tested during the day when 5 kg water was charged in the basin. The temperatures of the still glass cover (*T*_*g*_), absorber plate (*T*_*p*_), and basin water (*T*_*b-w*_) are hourly measured using a K-type thermocouple. The hourly productivity is collected in a graduated cone and weighted using a digital balance (accuracy 0.0001 g). The solar radiation (*I*) and surrounding air temperature (*T*_*a*_) are hourly measured using a prynometer and a thermometer. The second experiment was conducted when the LHSS was integrated with the SWH and the SBSS. In this case, the SBSS and the LHSS integrated with the SWH are separately operated from 8:00 am to 5:00 pm. Then, the LHSS is disconnected from the SWH and reconnected to the SBSS where the basin water is circulated between the SBSS and the LHSS from 5:00 pm to 8:00 am of the next day. Keep in mind that the hourly measurements of the system temperatures and productivity were taken until 11:00 pm. The temperature of water at the outlet of SWH (*T*_*o-h*_) and that of water in the storage tank (*T*_*sw*_) are also measured in this case. The temperature at different points of the PCM was regularly distributed with depth, and the redial axes were also measured using 5 K-type thermocouples. Then, the average value of the PCM temperature (*T*_PCM(av)_) was calculated. The accumulated productivity during the period from 11:00 pm to 8:00 am was measured at 8:00 am in the early morning on the next day.

## Results and discussions

### Development of the latent heat storage system

The LHSS was enhanced by using two techniques: first by adding CuO nanoparticles to the PW with different weight concentrations and then spiral finned tubes were used within the heat exchanger.

**Fig. 5:**
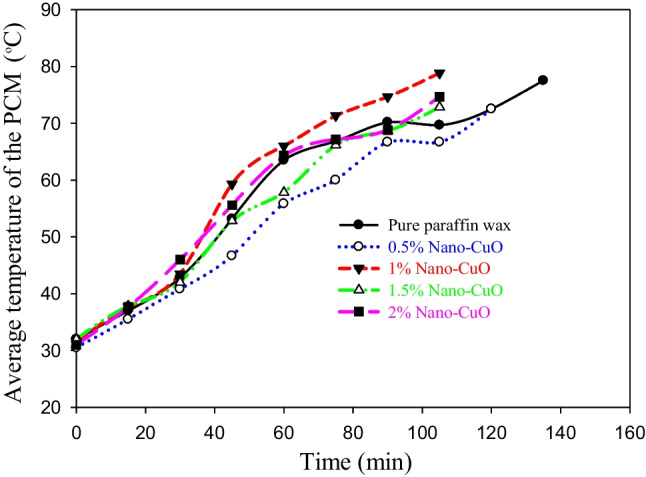
The effect of weight fraction concentration of CuO on the average temperature of the PCM during the melting process

**Fig. 6:**
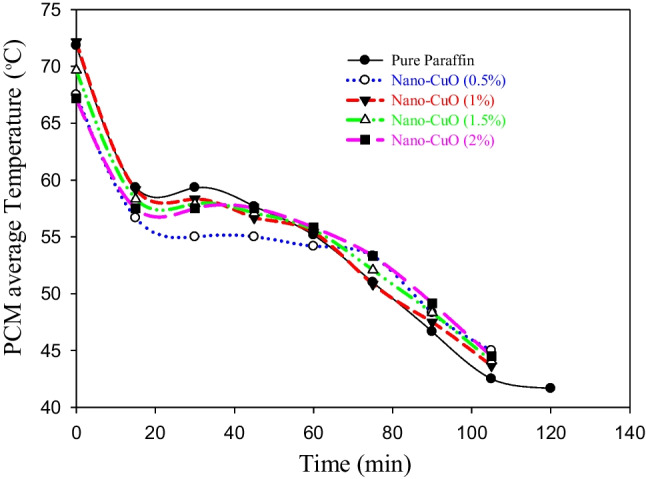
The effect of weight fraction concentration of CuO nanoparticles on the average temperature of the PCM during the solidification process

Figures 5 and 6 show the average temperature of the PCM during the melting and solidification processes, respectively. Keep in mind that all the experiments were continued until complete melting was achieved. It is plainly seen in Fig. 5 that adding the CuO nanoparticle to the PW with a weight fraction of 1% is the best among the tested concentrations as it not only achieves the lower melting time of the PCM but also provides higher PCM temperatures. The weight concentrations of 1.5 and 2% also achieve the same melting time but with a lower average PCM temperature. So, the weight fraction concentration of 1% is considered the optimal concentration from an economic point of view, where a low amount of nanoparticle was used. Similar results were obtained during the solidification process where the solidification time of 45 min was found in three cases: CuO was added by weight fractions of 1, 1.5, and 2% compared to 60 min in two cases: pure PW and CuO was added with a concentration of 0.5% as shown in Fig. 6. The solidification time is simply estimated by calculating the time of the flat part of the curves displayed in Fig. 6. These flat parts started, in all cases, when the average temperature of the PCM reaches 56 °C, which is the melting/solidification point of the PCM.

**Fig. 7:**
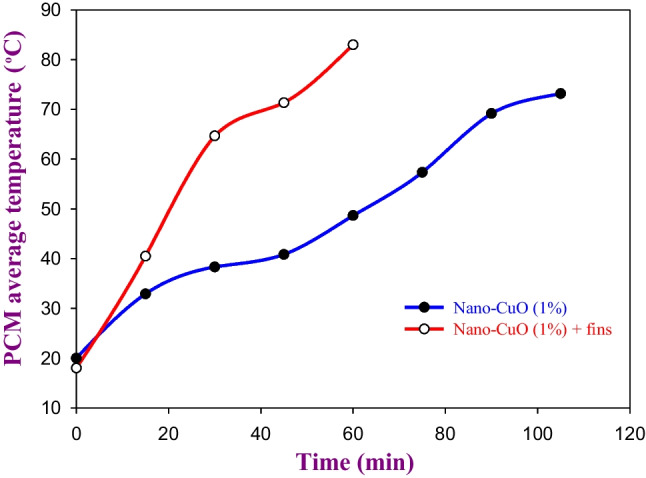
The effect of using spiral finned tubes within the LHSS on the melting time

**Fig. 8:**
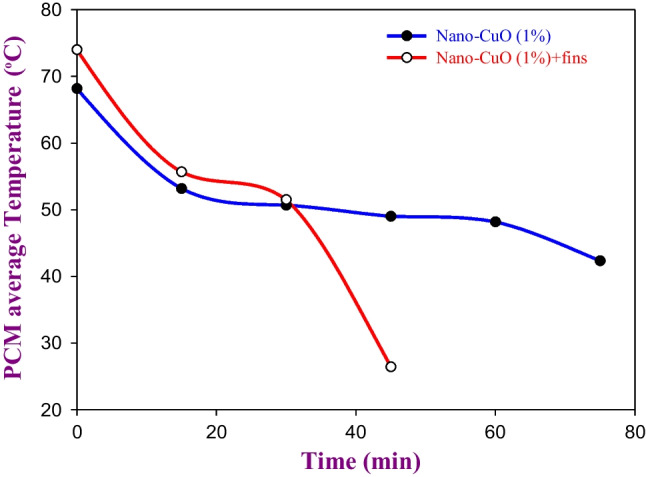
The effect of using spiral finned tubes within the LHSS on the solidification time

Figures 7 and 8 show the effect of using the spiral finned tube within the TSP on the average temperature of the PCM during the melting and solidification processes, respectively. The utilization of the spiral finned tubes within the TSP at the optimal concentration of nano-CuO not only reduces the melting time by 42.85% (from 105 to 60 min) but also significantly reduces the solidification time by 66.6% (from 45 to 15 min) as visibly shown in Figs. 7 and 8, respectively. Based on the results obtained from the indoor experiment where the TSP was tested, the LHSS was designed when the CuO nanoparticle was added to the PW with a weight fraction concentration of 1% and spiral finned tubes were used.

### The solar still integrated with the enhanced LHSS

**Fig. 9:**
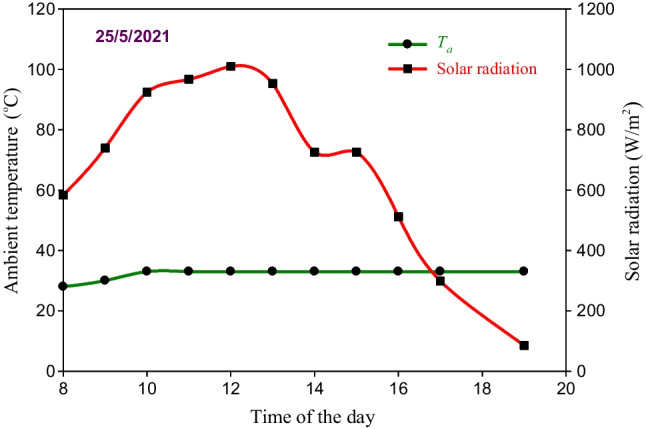
Climatic conditions measured on May 25, 2021

The solar intensity and environmental air temperature measured on May 25, 2021 are illustrated in Fig. 9. According to the results in Fig. 9, high values of *I* were measured between 9:00 am and 3:00 pm ranging from 725.5 to 1010 W/m^2^. The average value of solar intensity in this day is 684.1 W/m^2^, while the air temperature measured on this day varied from 28 to 33 °C during the period from 8:00 to 10:00 am and then remains constant at 33 °C until 7:00 pm.

**Fig. 10:**
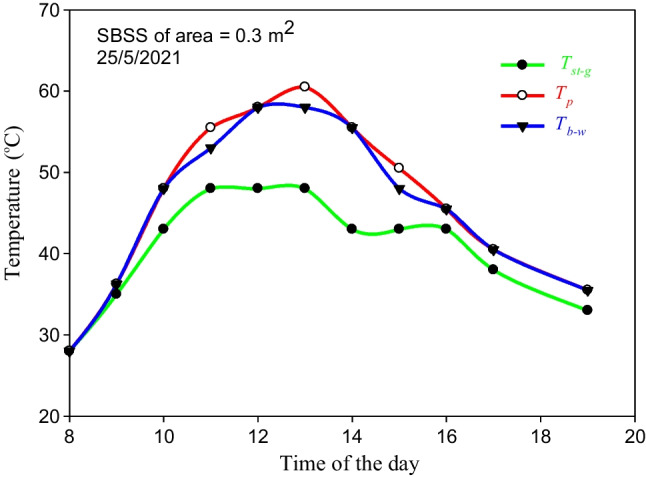
The hourly measured values of *T*_*st-g*_, *T*_*p*_, and *T*_*b-w*_ when the SBSS was tested on May 25, 2021

**Fig. 11:**
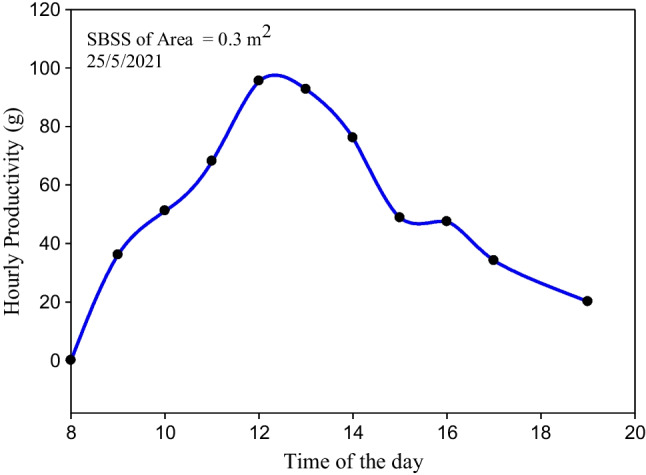
The hourly productivity of the SBSS without the storage system on May 25, 2021

The SBSS without using a storage system was tested on May 25, 2021 when 5 kg water was used in the basin. The temperatures of *T*_*st-g*_, *T*_*p*_, and *T*_*b-w*_ were hourly measured during the day long. The measured values of *T*_*st-g*_, *T*_*p*_, and *T*_*b-w*_ are displayed in Fig. 10. The maximum measured *T*_*p*_ and *T*_*b-w*_ are 60.5 and 58 °C, respectively. Based on the results in Fig. 10, high-temperature differences between the basin water and glass cover temperatures varying between 5 and 12.5 °C were found during the period from 11:00 am to 3:00 pm, as shown in Fig. 10. So, considerable high values of the hourly productivity are achieved during this period, as seen in Fig. 11. The low productivity of the SBSS after 3:00 pm shows the importance of using a suitable energy storage system. The fluctuation in the measured temperatures between 2:00 and 4:00 pm shown in Fig. 10 due to the cloudy weather and its negative effect on the hourly productivity shown in Fig. 11 assure the need of using an energy storage system.

**Fig. 12:**
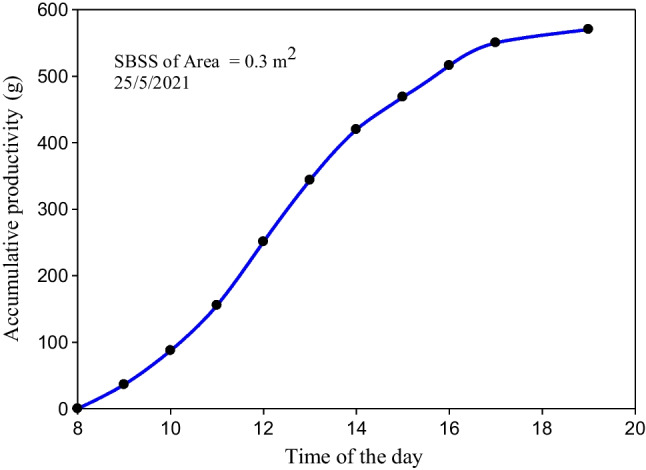
The accumulative productivity of the SBSS tested on May 25, 2021

The accumulative productivity of the SBSS tested on May 25, 2021 without the energy storage system is displayed in Fig. 12. The results showed that the accumulative productivity of the tested SBSS was 570.24 g after 10 working hours while the accumulative productivity during the night, which is measured at 8:00 am in the morning of May 26, 2021 was 141 g. The daily productivity of the still of 0.3 m^2^ was found to be 711.24 g/day (2370.8 g/day.m^2^), which is closes to the results obtained by Tabrizi et al. (Tabrizi et al. 2010) when their proposed stepped solar still was tested without a PCM. It is also considered relatively low compared to that obtained by Shalaby et al. when the v-corrugated absorber solar till is tested without a PCM. Keep in mind that in the current study, a flat absorber was used. In addition, still wall shadows are of negative significant effect in the current study as a small still area was used.

**Fig. 13:**
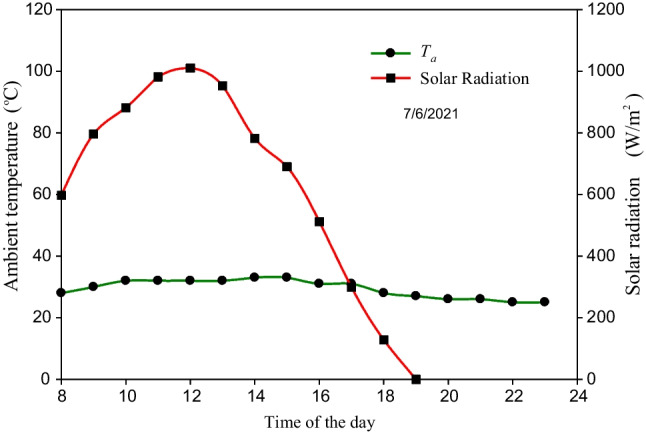
Climatic conditions measured on June 7, 2021

The SBSS was also tested on June 7, 2021 when it integrated with the LHSS and SWH. In this case, the SBSS and LHSS integrated with the SWH were separately operated from 8:00 am to 5:00 pm. Then, the LHSS is disconnected from the SWH and reconnected to the SBSS where the basin water is circulated between the SBSS and the LHSS from 5:00 pm to 8:00 am of the next day. Keep in mind that the hourly measurements of the system temperatures and productivities were taken until 11:00 pm while the accumulated productivity was measured at 8:00 am on the next day. The values of *I* and *T*_*a*_ measured June 7, 2021 are approximately similar to those measured during the testing of the SBSS without storage system (Fig. 9) as seen in Fig. 13. Where *T*_*a*_ varies between 28 and 33 °C during the sunshine period before dropping to reach 25 at 11:00 pm while the average value of *I* in this day is 635.9 W/m^2^.

**Fig. 14:**
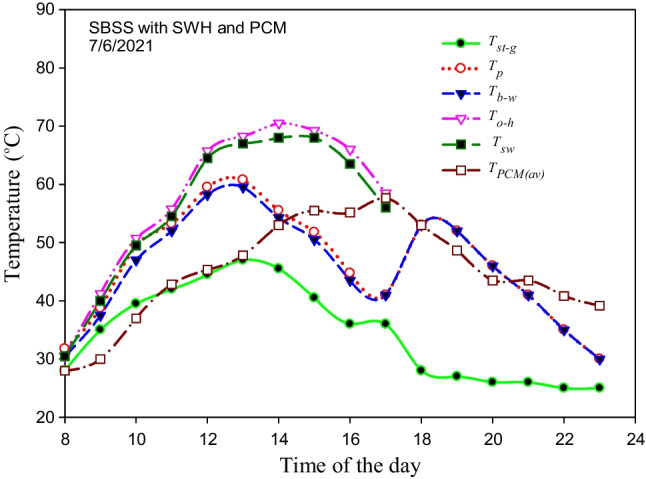
Variation in the measured temperature of several elements of the SBSS and the storage system

**Fig. 15:**
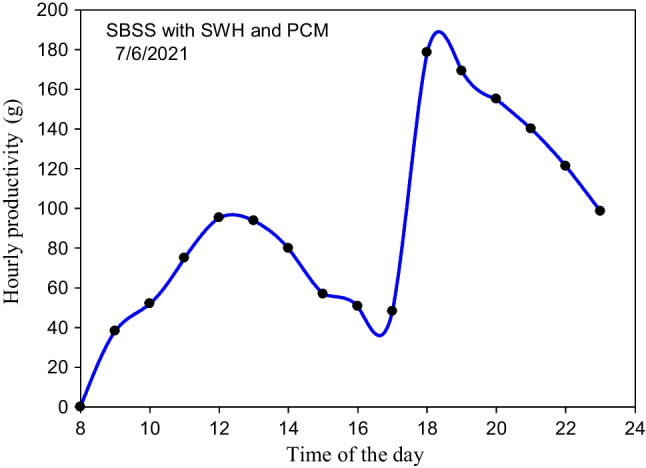
The hourly productivity of the SBSS integrated with the LHSS

In the case of integrating the SBSS with the storage system, the temperature of the different elements of the SBSS and storage system such as *T*_*st-g*_, *T*_*p*_, *T*_*b-w*_, *T*_*o-h*_, *T*_*sw*_, and *T*_PCM(av)_ were hourly measured and displayed in Fig. 14. The PCM completely melted between 4:00 and 5:00 pm when its average temperature exceeds the melting point (56 °C) as visibly seen in Fig. 14. After ensuing the complete melting of the PCM at 5:00 pm, the LHSS is disconnected to the SWH and reconnected to the SBSS where the stored energy is transferred from the LHSS to the basin water circulated between the still and the storage system. This explains the high increment in basin temperature (12 °C) after 1 h of integrating the LHSS with the still while the average temperature of the PCM drops to 53 °C, which means that the discharging time is less than 1 h. This large heat transferred to the basin water during the discharging process kept at an approximately constant basin water temperature (52–53 °C) during the period 6:00 to 7:00 pm. The previous result showed the positive effect of using both the CuO nanomaterial and the spiral finned tube within the LHSS as a very short discharging time was achieved. After this time, the remaining sensible heat stored in the PW is not enough to keep a constant basin water temperature for more time after 7:00 pm. So, the basin water temperatures starts to dramatically decrease with time to reach 30 °C at 11:00 pm. So, using several storage tanks is recommended to be integrated with the still one by one during the night period. The temperature difference between the basin water and the glass cover varies from 2.5 to 13.75 °C during the sunshine period compared to the 5 to 25 °C during the night when the LHSS was integrated with the SBSS where the system benefits from the drop that happened in the surrounding air temperature. This high difference (*T*_*b-w*_*-T*_*st-g*_) explains the high productivity achieved during the night when the storage system was used, as seen in Fig. 15. The maximum hourly productivity (178.5 g) is achieved at 6:00 pm flowed by 169.2 g at 7:00 pm when the basin water temperature is between 52 and 53 °C. After that time, the hourly productivity decreases with the passage of time to reach 98.6 g at 11:00 pm, as illustrated in Fig. 15.

**Fig. 16:**
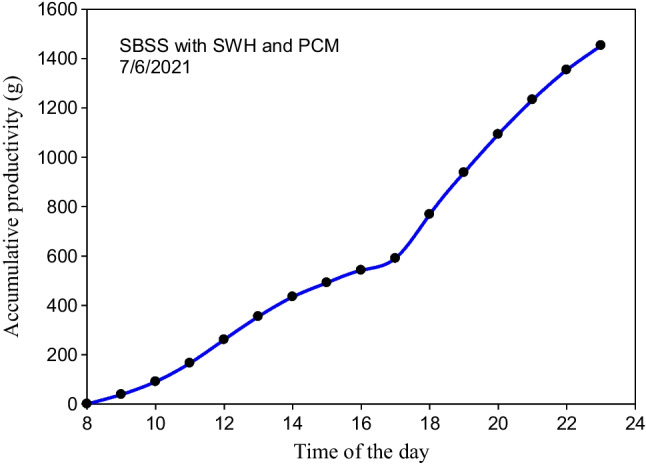
Accumulative productivity of the SBSS integrated with the LHSS

The accumulative productivity of the SBSS integrated with the LHSS is illustrated in Fig. 16. The accumulative productivity from 8:00 am to 11:00 pm was 1452.8 g, while it was 530 g during the period from 11:00 pm (June 7) to 8:00 am (June 8, 2021). Based on these results, the daily productivity of the system consisting of the SBSS of 0.3 m^2^ and the SWH with an area 0.5 m^2^ is 1982.8 g while the daily productivity per square meter of the solar field is 2478.5 (g/day.m^2^). So, an improvement in daily productivity of 4.54% was achieved when the LHSS was integrated with the SBSS. Although this improvement is low compared to that obtained when the PCM was used under the still basin absorber plate, which reaches to 12% (Shalaby et al. 2016), the separation between the storage system and the SBSS avoids the risk of PCM leakage with the passage of time.

## Economic analysis

In this section, we analyzed the cost per liter of the fresh water produced from the SBSS in two cases: first without the LHSS and then when the LHSS is integrated with the system. The cost is affected by the several parameters given in Table 1 (Fath et al. 2003).where *i* is the interest per year (%/year), which is assumed to be 12%/year, *n* is the number of life years, which is assumed to be 10 years, *P* is the capital cost ($), *ECPD* is the electrical consumption per day (kWh), *EC* is the household electrical cost ($/kWh), and *AP* is the annual productivity (*l*). Table 2 shows the cost of the several parts of the SBSS and the storage system ($1 = 15.68 LE). Assuming that the system was operated for 300 days/year while the remaining days are specified for maintenance (Shalaby et al. 2016), the different costs were calculated and summarized in Table 3.

**Table 1:** The several parameters affecting the estimated cost

	The parameter	Equation
1	The sinking fund factor (*SFF*)	$$SFF=i/\left[{(1+i)}^{n}-1\right]$$
2	*CRF* is the capital recovery factor (*CRF*)	$$CRF=(SFF)\times {(1+i)}^{n}$$
3	The fixed annual cost ($) (*FAC*)	$$FAC=P\times (CRF)$$
4	The scrap value ($) (*S*)	$$S=0.2\times P$$
5	The annual scrap value ($) (*ASV*)	$$ASV=(SFF)\times S$$
6	The annual maintenance operational cost ($) (*AMC*)	$$AMC=0.15\times FAC$$
7	The annual electricity cost ($) (*AEC*)	$$AEC=ECPD*EC*300$$
8	The annual cost ($) (*AC*)	$$AC=FAC+AMC+ACEEC-ASV$$
9	The cost of distilled water per liter ($/*L*) (CPL)	$$CPL=AC/AP$$

**Table 2:** The costs of several parts of the two proposed systems

The component	The cost ($)
The SBSS	191.32
The SWH	82.9
The LHSS	63.77
Pump	22.32
Connecting tubes and valves	31.88
Total direct cost of the SBSS and LHSS	392.19

**Table 3:** The calculated values of several parameters

The parameter	The SBSS	The SBSS with SWH and LHSS
*SFF*	0.057	0.057
*CRF*	0.177	0.177
*FAC ($)*	33.86	69.41
*S* ($)	38.26	78.43
*ASV($)*	2.18	4.47
*AMC($)*	5.07	10.41
*AEC($)*	0	77.07
*AC($)*	36.75	152.42
CPL *($/l)*	0.171	0.25

The calculated values of the CPL of the SBSS with and without the LHSS were found to be 0.25 and 0.171 $/L, respectively. These values are close to the results found by Rahbar and Esfahani (0.2696 $/L) (Rahbar and Esfahani 2012), Esfahani et al. (0.1792 $/L) (Rahbar and Esfahani 2011), Kumar and Tiwari (0.1875 $/L) (Kumar and Tiwari 2009), and Abdallah and Badran (0.1667 $/L) (Abdallah and Badran 2008), while these costs are considered very high compared to other literature studies (0.022–0.1103) (Kabeel et al. 2014; Gorjian et al. 2014). This difference in cost estimation is based on the capacity (or the area) of the studied solar still, and as the capacity increases, the cost per liter decreases. This explains the high CPL obtained in this study where the projected area of the SBSS was only 0.3 m^2^. The high value of the CPL was found when the LHSS was used mainly due the high consumed power where a 0.35-HP pump was used. Actually, a low-power pump may be used for this small capacity system. In other words, the used pump of the lowest power found in Egypt market, perfectly appropriate a higher capacity system up to 10 times area of the studied system. 

## Conclusions

The single-basin solar still is constructed and tested when it is connected to the LHSS and free of the LHSS. The outdoor testing results showed that:

- The proposed design of the LHSS has succeeded to overcome all challenges associated with using paraffin wax as storage material with the SBSS.
- An improvement in daily productivity of 4.54% was achieved when the LHSS was integrated with the SBSS.
- The highest hourly productivity is directly achieved 1 h after circulating the basin water between the basin and the LHSS.
- The calculated values of the CPL of the SBSS with and without the LHSS were found to be 0.25 and 0.171 $/L, respectively. These high costs will be significantly reduced when a large-scale system was installed.
- Using several storage tanks is recommended to be integrated with the SBSS one by one during the night period to increase the operating time and fresh water productivity.
- It is also recommended to test several types of PCM enhanced by high conductive nanomaterials.
- The effect of using solar reflectors with the SWH and the SBSS is also recommended for future works.

## Data Availability

The datasets generated during and/or analyzed during the current study are available from the corresponding author upon reasonable request.

## Nomenclatures

*I*: Solar intensity (W/m^2^)
PCM: Phase change material
SBSS: Single-basin solar still
SWH: Solar water heater
*T*_*a*_: Ambient temperature (°C)
*T*_*b-w*_: Temperature of basin water (°C)
*T*_*o-h*_: Outlet temperature of the SWH (°C)
*T*_*p*_: Temperature of the basin absorber plate (°C)
*T*_PCM(av)_: PCM average temperature (°C)
*T*_*st-g*_: Temperature of the solar still glass cover
*T*_*sw*_: Temperature of water in the storage tank (°C)

## References

[CR1] Abdallah S, Badran OO (2008). Sun tracking system for productivity enhancement of solar still. Desalination.

[CR2] Abdallah S, Abu-Khader MM, Badran O (2009). Effect of various absorbing materials on the thermal performance of solar stills. Desalination.

[CR3] Akash BA, Mohsen MS, Osta O, Elayan Y (1998). Experimental evaluation of a single-basin solar still using different absorbing materials. Renew Energy.

[CR4] Ansari O, Asbik M, Bah A, Arbaoui A, Khmou A (2013). Desalination of the brackish water using a passive solar still with a heat energy storage system. Desalination.

[CR5] Arunkumar T, Denkenberger D, Ahsan A, Jayaprakash R (2013). The augmentation of distillate yield by using concentrator coupled solar still with phase change material. Desalination.

[CR6] Elbar ARA, Hassan H (2019). Experimental investigation on the impact of thermal energy storage on the solar still performance coupled with PV module via new integration. Solar Energy.

[CR7] El-Sebaii A, Shalaby S (2015). Parametric study and heat transfer mechanisms of single basin v-corrugated solar still. Desalination Water Treat.

[CR8] Fath HES, El-Samanoudy M, Fahmy K, Hassabou A (2003). Thermal-economic analysis and comparison between pyramid-shaped and single-slope solar still configurations. Desalination.

[CR9] Gorjian S, Ghobadian B, Tavakkoli Hashjin T, Banakar A (2014). Experimental performance evaluation of a stand-alone point-focus parabolic solar still. Desalination.

[CR10] Hammad FA, Shalaby SM, Kabeel AE, Zayed ME (2022). Experimental investigation and thermo-economic performance analysis of a modified solar distiller design with thermal storage material and v-corrugated absorber basin. J Energy Storage.

[CR11] Hedayati-Mehdiabadi E, Sarhaddi F, Sobhnamayan F (2020). Exergy performance evaluation of a basin-type double-slope solar still equipped with phase-change material and PV/T collector. Renew Energy.

[CR12] Kabeel AE, Abdelgaied M (2017). Observational study of modified solar still coupled with oil serpentine loop from cylindrical parabolic concentrator and phase changing material under basin. Solar Energy.

[CR13] Kabeel AE, Omara ZM, Essa FA (2014). Improving the performance of solar still by using nanofluids and providing vacuum. Energy Convers Manag.

[CR14] Kalidasa Murugavel K, Sivakumar S, Riaz Ahamed J, Chockalingam KKSK, Srithar K (2010). Single basin double slope solar still with minimum basin depth and energy storing materials. Appl Energy.

[CR15] Kumar S, Tiwari GN (2009). Life cycle cost analysis of single slope hybrid (PV/T) active solar still. Appl Energy.

[CR16] Nafey AS, Abdelkader M, Abdelmotalip A, Mabrouk AA (2001). Solar still productivity enhancement. Energy Convers Manag.

[CR17] Naim MM, Abd El Kawi MA (2003). Non-conventional solar stills part 1. Non-conventional solar stills with charcoal particles as absorber medium. Desalination.

[CR18] Okeke CE, Egarievwe SU, Animalu AOE (1990). Effects of coal and charcoal on solar-still performance. Energy.

[CR19] Omara ZM, Hamed MH, Kabeel AE (2011). Performance of finned and corrugated absorbers solar stills under Egyptian conditions. Desalination.

[CR20] Omara ZM, Kabeel AE, Younes MM (2013). Enhancing the stepped solar still performance using internal reflectors. Desalination.

[CR21] Panchal H (2022). Performance evaluation of using evacuated tubes solar collector, perforated fins, and pebbles in a solar still – experimental study and CO2 mitigation analysis. Environ Sci Pollut Res.

[CR22] Rahbar N, Esfahani J (2011) Utilization of thermoelectric cooling in a portable active solar still--an experimental study on winter days. Desalination 269. 10.1016/j.desal.2010.10.062

[CR23] Rahbar N, Esfahani JA (2012). Experimental study of a novel portable solar still by utilizing the heat pipe and thermoelectric module. Desalination.

[CR24] Sakthivel M, Shanmugasundaram S (2008). Effect of energy storage medium (black granite gravel) on the performance of a solar still. Int J Energy Res.

[CR25] Sarhaddi F, Farshchi Tabrizi F, Aghaei Zoori H, Mousavi SAHS (2017). Comparative study of two weir type cascade solar stills with and without PCM storage using energy and exergy analysis. Energy Convers Manag.

[CR26] Shalaby S (2017). Reverse osmosis desalination powered by photovoltaic and solar Rankine cycle power systems: a review. Renew Sustain Energy Rev.

[CR27] Shalaby S, El-Bialy E, El-Sebaii A (2016). An experimental investigation of a v-corrugated absorber single-basin solar still using PCM. Desalination.

[CR28] Shama A, Kabeel AE, Moharram BM, Abosheiasha HF (2021). Improvement of the thermal properties of paraffin wax using high conductive nanomaterial to appropriate the solar thermal applications. Appl Nanosci.

[CR29] Shanmugan S, Rajamohan P, Mutharasu D (2008). Performance study on an acrylic mirror boosted solar distillation unit utilizing seawater. Desalination.

[CR30] Shoeibi S, Mirjalily SAA, Kargarsharifabad H, Panchal H, Dhivagar R (2022). Correction to: Comparative study of double-slope solar still, hemispherical solar still, and tubular solar still using Al2O3/water film cooling: a numerical study and CO2 mitigation analysis. Environ Sci Pollut Res.

[CR31] Tabrizi FF, Dashtban M, Moghaddam H (2010). Experimental investigation of a weir-type cascade solar still with built-in latent heat thermal energy storage system. Desalination.

[CR32] Tanaka H (2009). Experimental study of a basin type solar still with internal and external reflectors in winter. Desalination.

[CR33] Tiwari GN, Suneja S (1998). Performance evaluation of an inverted absorber solar still. Energy Convers Manag.

[CR34] Velmurugan V, Gopalakrishnan M, Raghu R, Srithar K (2008). Single basin solar still with fin for enhancing productivity. Energy Convers Manage.

[CR35] Velmurugan V, Deenadayalan CK, Vinod H, Srithar K (2008). Desalination of effluent using fin type solar still. Energy.

[CR36] Yousef MS, Hassan H (2019). An experimental work on the performance of single slope solar still incorporated with latent heat storage system in hot climate conditions. J Clean Prod.

[CR37] Yousef MS, Hassan H (2019). Energetic and exergetic performance assessment of the inclusion of phase change materials (PCM) in a solar distillation system. Energy Convers Manag.

[CR38] Zayed ME (2019). Applications of cascaded phase change materials in solar water collector storage tanks: A review. Solar Energy Mater Solar Cells.

